# Humor of the Leader: A Source of Creativity of Employees Through Psychological Empowerment or Unethical Behavior Through Perceived Power? The Role of Self-Deprecating Behavior

**DOI:** 10.3389/fpsyg.2021.635300

**Published:** 2021-09-03

**Authors:** Hassan Ali, Asif Mahmood, Ayyaz Ahmad, Amir Ikram

**Affiliations:** ^1^Institute of Business and Management, University of Engineering and Technology, Lahore, Pakistan; ^2^Department of Business Studies, Namal Institute, Mianwali, Pakistan; ^3^Institute of Quality and Technology Management, University of the Punjab, Lahore, Pakistan

**Keywords:** leader's sense of humor, leader-member exchange, employee creativity, psychological empowerment, leader's self-deprecating humor, perceive power, unethical behavior

## Abstract

Although we use humor in our daily communication, there still needs to cognize its effects on the attitudes and behavior of the employees. Based on benign violation theory (BVT), the study proposes that leader's humor (LH) conveys social information about counter norms. The BVT has been amalgamated with social information processing theory (SIPT) to develop hypotheses assuming the consequences of LH on the attitude and behavior of the employees. This study hypothesizes that even though LH is linked positively with employee creativity *via* leader-member exchange and psychological empowerment in sequence (blessing path), it may also send information to the employees about the acceptability of norm violation. This perception ultimately leads to power perception and, causes unethical behavior in the series (curse path). Moreover, this study also postulates that leader's self-deprecating humor (LSDH) moderates these indirect effects by enhancing the blessing and reducing the curse, which emerged from LH. Quantitative data of 630 software engineers from software houses based in Pakistan provided support to test the hypotheses. The results demonstrate that LH is a double-edge sword that enhances blessing (creativity) as well as curse (employee unethical behavior), whereas LSDH augments the blessing and throttles back the curse. Theoretical and managerial implications have also been discussed.

## Introduction

We use humor in daily communication; therefore, the critical aspects of humor play an important role in determining how we form beliefs about others (Bitterly and Schweitzer, 2019). Humor is a broader concept defined as the ability of an individual to amuse others (Martin et al., 2003). Due to this amusement at the workplace, employees have the freedom in shaping their behavior. Some employees use this freedom in the wrong way (i.e., curse), while some take it in a positive sense for building up their behavior (i.e., blessing) (Yam et al., 2018; Kong et al., 2019). It is evident that organizations seek blessings, which are important for making organizations lucrative, and organizations also want to overcome curses, which are vicious for the existence of organizations.

Overall, this brief description of humor indicates that it is a blessing as well as a curse for an organization. For example, the humor of a manager triggers positive emotions only when subordinates perceive it positively and vice versa (Wijewardena et al., 2017). Furthermore, leader humor (LH) predicts work engagement and follower deviance (Yam et al., 2018), the intent of the employee to stay, job performance (Kong et al., 2019), employee advocacy (Karakowsky et al., 2020). It is also associated with job performance, organizational citizenship behavior (OCB), job satisfaction, affective organizational commitment, intent to stay, and positive emotions *via* leader–member exchange (LME or LMX) relationship (Kong et al., 2019). Besides LH, some researchers find blessing and curse through other mechanisms, such as power, which predicts unethical behavior (UB) (Yap et al., 2013; Dubois et al., 2015; Rees et al., 2019), and psychological empowerment (PE), which predicts employee creativity (Javed et al., 2017). The researchers provided the causes of blessings and curses but failed to create a comprehensive framework in double-edged sword form linking LH to creativity and UB in parallel. Moreover, research on humor is also unable to control negativity that emerges from LH. So, this is the first study for considering these neglected mechanisms.

Thus, it is posited in this study that the sense of LH could be a double-edged sword. The blessing is represented by a path from the sense of LH to employee creativity through the sequential mediating role of LMX and PE. In contrast, the curse is represented by a path from the sense of LH to UB through the sequential mediating role of perceived acceptability of norm violation (PANV) and perceived power. Moreover, it is presumed that LSDH will moderate the effects so that it will reduce the curse and enhance the blessing that emerges from LH.

This study is important for many reasons. First, literature on humor highlighting its blessing and curse in organizational settings is still scarce (Yam et al., 2018; Kong et al., 2019), characterizing it as “sporadic” in organizational research (Robert et al., 2015). So, this research contributes as an empirical study in extending the literature on LH in a work setting. From a practical point of view, this study is important for all leaders or managers because it would help understand the drivers of creativity and unethical behavior in the workplace. The first of these is necessary to keep the organization alive and the latter is imperative to make the organization attractive for work. In fact, LH is a low-cost strategy compared to other structural policies for enhancing positive outcomes (e.g., the performance, creativity, and work engagement of the employee). So, from this point of view, research on LH is valuable for organizations. Furthermore, this research is based on the integration of benign violation theory (BVT) and social information processing theory (SIPT). The BVT was developed by McGraw and Warren (2010). It was applied to study the topics like LH (Yam et al., 2018), eliciting mixed, positive, and negative emotions from the video library (Samson et al., 2016). This theory indicates that humor is basically a cause of norm violations. However, these violations must be benign. The BVT applied to this study suggests what humor is. However, another theory is required to know the consequences of humor. Therefore, SIPT has been used for this purpose. The main tenet of SIPT is that employees get signals about what behavior is rewarded and what is punished from social information. This theory was used to examine the issues like the relationship between cues and malevolent creativity (Gutworth et al., 2018); moreover, how a real leader acts as a source of information to promote the input of the employee in the workplace (Boekhorst, 2015). So, both these theories have been combined for this study.

## Literature Review

The literature on LH has been reviewed to know the current state of knowledge and identify the gaps. In this context, different researchers contributed to LH topics in recent years. Among these contributions, Karakowsky et al. (2020) posited that humor is a valuable leadership characteristic. Based on 304 employees and their leaders working in large Canadian retail stores, this research shows that LH can ‘affect the feedback-seeking behavior of the employees through cognition and affective-based trust. Besides it, Yam et al. (2018) stated that the organizations might have to bear costs with benefits due to LH. This research also provides that leaders should get training about how to use humor to reap benefits because humor is a low-cost strategy for workplace engagement compared to other structural policies. Moreover, Cooper et al. (2018) indicate that LH is an interpersonal resource. These researchers provide that their work is first to establish after empirically testing a link between LH and organizational citizenship behavior (OCB). Another research study also extended the literature by highlighting that LH significantly predicts intent to stay, OCB, job satisfaction, affective organizational commitment, and job performance *via* LMX and positive emotions (Kong et al., 2019). It suggests that those employees are more innovative who perceive their leader as more humorous. Beside these, the perceived innovative climate is not moderating the impact of LH on innovation (Pundt, 2015). In contrast, being male, high-status employees and aged are linked with greater punishment when displaying inappropriate behavior (Sacco et al., 2020). Moreover, trust in a leader enhances the relationship between LH and inclusion perception (Tremblay, 2016). This phenomenon is the opposite when leader-member has hostile relations. Goswami et al. (2016) revealed that LH is not linked with OCB and performance *via* positive emotions.

On the other hand, tenure with a leader enhances the association between affiliative humor and leader–employee relations (Robert et al., 2015). Moreover, the transformational style enhances the relationship between LH and positive emotions. Pundt and Venz (2017) highlighted that LH reduces disengagement and improves affective commitment *via* leader–member exchange. Whereas, LH has no influence on emotional exhaustion *via* LMX. Gkorezis and Bellou (2016) contributed that trust in a leader partially mediates the relationship between leader's self-deprecating humor of a leader and his effective perception. Alongside these studies, Pundt and Herrmann (2015) posited that identification with a leader mediates the effect of affiliative LH on LMX. But identification with a leader does not mediate the influence of aggressive humor on LMX. Moreover, Gkorezis et al. (2014) suggested that the positive humor of the leader predicts organizational cynicism *via* LMX. The major contribution of this research is that it is the first to test a link between the positive humor of the leader and organizational cynicism *via* LMX. Further, Thelen (2019) concluded, based on a quantitative survey from 350 employees working in different organizations, that supervisor's humor style affects employee's advocacy by building a connection between the humor style of a supervisor and employee advocacy through the mediating role of supervisor authenticity and the organizational relationship of the employee in sequence. Moreover, Hu and Luo (2020) demonstrated that LH is positively linked with employee creativity through the task resource of the employee and his commitment to organization while taking the perspective of the employee as a moderator in the relationship between LH based on time-lagged data from 358 employees and leaders of hi-tech companies located in China.

Additionally, literature establishes that blessing like creativity is a consequence of variables like psychological empowerment (Javed et al., 2017; Yang et al., 2019). On the other side, curses, such as UB is predicted by variables like power (Yap et al., 2013; Dubois et al., 2015; Rees et al., 2019). Besides, these norm violations also predict power, and power is mainly considered a curse (power is corrupt) (Van Kleef et al., 2011). Moreover, it is seen that the self-deprecating humor used by Obama during his election campaign in 2008 has an impact on engaging followers (Stewart, 2011). So, from this research, it can be said that self-deprecating humor is also a driver of blessing.

Moreover, this research is based on the integration of two theories: SIPT and BVT. The SIPT was developed by Salancik and Pfeffer (1978), which indicated that employees get information from the social environment, and make meaning of it after processing. In other words, employees build up their attitude and construct their perception grounded on social cues and information, which affects their behavior. Copeland (1994) posited that prominent sources for driving social information are those who have high status. Another research study highlighted that the leader is a source for transmitting information to employees (Boekhorst, 2015). The understanding of the employees with regard to the environment depends upon cues that emerge from the behavior of the leader, and the employees set their cognitions that best suit the environment (Jibao et al., 2018). It was also found that the behavior of the leader is a forceful source in shaping the attitude and behavior of the employees, and a symbol of power to influence the attitude of the employees (Xiaoxiao and Shi, 2015). A research study specified that social cues impinge malevolent creativity, intentionally harming others with creative thinking (Gutworth et al., 2018). Grounded on SIPT, a research study postulated how servant leaders influence the emotional labor of the employees. Data were collected from 81 working units of a food company based in China. The results show that servant leadership positively affects deep acting while it has a negative impact on surface-acting. Moreover, this study also revealed that servant leadership influences the emotional labor of the employees through affective trust rather than cognitive trust (Lu et al., 2019).

Another research study suggests that ethical leadership enhances performance by mediating creative self-efficacy based on SIPT. Data were collected from 512 employees working in service industries in Ghana. The results highlighted that ethical leadership influences performance through the mediating role of creative-self efficacy (Wadei et al., 2020). It was also indicated that the humility of the leader affects team innovation by drawing upon SIPT. Data from 90 teams showed that leader humility positively affects team innovation through team voice climate. Moreover, task interdependence plays a role of moderating variable in the relationship between leader humility and team innovation (Liu et al., 2017). In addition, research was conducted on how the information-seeking attitude of the leader impacts team innovation and performance. This research was based on SIPT to formulate that the information-seeking attitude of the leader has a relationship with team performance and innovations through the mediating role of team reflexivity. Moreover, cooperative outcome interdependence moderates the relationship between the information-seeking attitude of the leader and team reflexivity. Data were collected from pharmaceutical companies operating in China, and the sample size comprised 253 team members from 83 work teams. The results conveyed that the information-seeking attitude of the leader positively relates to team innovation and team performance *via* team reflexivity. Further, the outcomes of cooperative interdependence moderates the link between leader information seeking and team reflexivity, such that the relationship is stronger when cooperative outcomes interdependence high and weaker when it is low (Wang et al., 2020). Moreover, Kuenzi et al. (2019) drew their research on SIPT and social learning theory to conceptualize that employees exhibit less UB (behavior is not rewarded) because of ethical climate (social information). Moreover, Rego et al. (2017) suggested, based on SIPT, that employees experience the humility of the leader by social interaction (social information), and this humility of the leader is positively linked with team performance (behavior is rewarded). In line with this evidence, we suggest that LH is a source of social information.

On the other hand, McGraw and Warren (2010) proposed BVT, suggesting what humor is and what humor is not. This theory explains that humor entails benign norm violations and three conditions must be satisfied for things to be humorous. First, the violation should occur. Veatch (1998) indicated that violations have a variety of forms. For example, Gervais and Wilson (2005) posited that apparent physical threat, like in play fighting, is the origin of humor. With the evolution of humor, the situations expanded from physical threat to a variety of other forms for eliciting humor, including linguistic norms (Malapropism or unusual accents), personal dignity (physical deformities and slap stick), moral norms (disrespectfulness and bestiality), and social and cultural norms (strange behavior). Second, violations ought to be benign. It means, norms violation must be taken place, and it should be nonthreatening or benign. For example, people feel humorous and laugh when a loved person tickles them rather than a stranger. With these, norm violations should not be offensive or threatening in nature. Third, these first two conditions should occur simultaneously (i.e., norm violations should occur, and it must be benign). So, the BVT hypothesizes that anything perceived as violated norms seems humorous only if the violation is benign (McGraw and Warren, 2010).

Bettenhausen and Murnighan (1985) asserted that humor entails norm violations and signals to employees that violation of norms has social acceptance during interpersonal interactions. It is very relevant in organizations, a highly social environment with norms to be learned, conveyed, and practiced. Peter et al. (2012) proposed that humor is often beneficial and ubiquitous, and participants find things funny because of the benign nature of violations when they are hypothetically, socially, temporally, or spatially distant. Another research strengthened by six studies that employ social interaction, consumer product, and entertainment as stimuli indicated that BVT is better in hypothesizing the difference between humor and what is not than incongruity theories (Warren and McGraw, 2016). By drawing on BVT, it was indicated that morality impinges on humor, and tension exists between these two. It is because ethical leadership does not involve moral violations, and a leader with fewer moral violations has more trust (Yam et al., 2019). Further from the perspective of BVT, complainers are less likely to get sympathy when they complain in a humorous way (Peter et al., 2015). Moreover, Yam et al. (2018) used BVT to propose that LH often sends counter normative information to followers.

## Theory and Hypotheses Development

### Role of SIPT and BVT in Contribution to the Formulation of Research Framework

In order to formulate hypotheses, two theories, namely, BVT and SIPT have been considered. BVT provides that humor entails violations of formal or informal rules (norms). However, to understand the consequences of humor in the workplace, this theory should be integrated with a distinct theory to the vigorous inherent in organizational settings (Heath and Sitkin, 2001). For this purpose, SIPT has been taken, which argues that employees act consistently with the expectations and rules of their organization by processing the social cues. According to SIPT, employees perceive their leader as a role model, a guide to act in different conditions (Boekhorst, 2015). Thus, the leader sends implicit cues or information to subordinates, and they learn what behavior is demanded (i.e., punishable or rewarded) by processing this social information.

Further, employees make cognitive representation from the act of the leader, as a signal of values and expectations of work setting, suggested by SIPT. Such cognitive representations are not specific but general and symbolic. James et al. (1978) argued that employees try to develop the meaning of social information in a specific situation by looking toward the action of the person who has the highest status as gestalt representation, a fundamental principle, to apply across multiple situations. So, it can be concluded from these explanations that the behavior of the leader symbolizes a guide.

Thus, when amalgamated with BVT, SIPT provides that when a leader uses humor in the workplace, the consequences are more than simple mimicry (employees get that humor is rewarded and expected, rather than punishable). Instead, implicit information is sent that acting counter-normatively is acceptable, an expected way for doing things. Thus, it is argued here that when a leader uses humor in interaction with employees, it conveys two implicit messages. The first is to make counter norms acceptable (acceptability of norm violation), leading to deleterious effects of UB through perceived power. The second is a permissive exchange relationship between the leader and employees (leader–member exchange), which may positively impact the involvement of employees in creativity through psychological empowerment. Thus, the integration of BVT and SIPT indicates that when a leader uses humor (in a sense that is violating norms), it gives signals in two ways. First, employees process these signals through experience, and perceive that leader is vulnerable, permissive, and wants to reduce hierarchical distance. Hence, employees accept counter normative behavior, building up high-quality relations (*link from LH to leader–member exchange*). Thus, employees attributed to psychological empowerment as a result of the efforts of the leader to build up a strong relationship because employees perceive that they have a specialty which other employees do not have, that is why the leader wants a high-quality relationship with them *(link from LH to PE through leader–member exchange)*. Hence, this empowerment helps build a positive attitude, which is creativity, as employees think that this attitude is rewarding *(link from LH to employee creativity through LMX and PE in sequence)*. Second, employees process these signals through experience, and perceive the acceptability of norm violations because their role model is also violating the norms using humor-benign norm violations *(link from LH to acceptability of norm violation)*. In this way, acceptability of norm violation is attributed to perceived power-violators, who think that by violating norms, they look to be powerful *(link from LH to perceive power through acceptability of norm violation)*. Similarly, powerful nature leads toward UB-power is corrupt *(relation from LH to UB through perceiving power and acceptability of norm violation in sequence)*. Based on these arguments, the integration of these theories is relevant in constructing the following hypotheses.

### Implications for LMX, PE, and Employee Creativity

Leader–member exchange relationship defines status in leader–member relationships and is a social exchange process (Graen and Uhl-Bien, 1995). Basically, LMX depends upon mutual trust and respect (Mahsud et al., 2010). In the next paragraph, the integrational effects of both BVT and SIPT theories will be discussed to develop the relationship between LH and LMX.

The integration of both the theories postulates a positive association of LH with LMX variable in three ways. First, LH decreases the social distance of a leader with a member (Mesmer-Magnus et al., 2012). With LH, it seems a leader approves to violate the hierarchical system, and increases relationships by reducing the distance. Second, LH sends an implicit message that the leader is nonrestrictive, accepting averse normative behavior, signaling that the relationship of the leader is open, complicated, and playful with a specific employee. In fact, a research study in behavior ethics indicates that moral leaders do not allow adverse normative behavior, which is why they are less friendly and more restrictive (Wellman et al., 2016). Third, the LH sends a message that the leader likes to be vulnerable because the leader is violating norms, looking more open and less guarded during social interaction with their employees. Subordinates who find their leaders permissive, vulnerable, and de-emphasizing hierarchy may see that they are more relationship-oriented. Empirically, LH is associated positively with LMX (Yam et al., 2018). So, it can be suggested that a LH positively impacts building strong relations with their followers.

*H*_1_*; LH is positively linked with LMX*.

The effects of LH on LMX suggest here that LH is likely to enhance PE. PE can be defined as an increased intrinsic task motivation manifested in a set of four cognition reflecting the orientation of an individual to his or her work role: competence, impact, meaning, and self-determination (Spreitzer, 1995). These four concepts fit well to define psychological empowerment (Seibert et al., 2011). Moreover, all the four above-listed concepts are distinct; so researchers have no limitation to use four or one (Walumbwa and Hartnell, 2011; Kim and Beehr, 2017). But in this research, all the four concepts were used.

As members have a good quality relationship with the leader because of LH, they feel more empowered. The key of this postulate is good quality leader–member relations. Employees feel more empowered due to high-quality LMX (Wang et al., 2016) and this LMX is facilitated by leader behavior (Gu et al., 2013). Moreover, the combination of BVT and SIPT also indicates that LH gives employees implicit signals that the leader accepts counter normative behavior for building up a high-quality relationship. Thus, it leads to changing the attitudes of the employees like understanding the feeling of the boss and perceiving themselves more empowered (Gkorezis et al., 2011). Thus, it can be concluded that LH positively drives empowerment *via* high-quality LMX.

*H*_2_*; LH is positively linked with psychological empowerment via leader–member exchange*.

Through LH effects on psychological empowerment, mediated by LMX, it suggests that LH is likely to enhance employee creativity. Withagen and van der Kamp (2018) defined creativity as discovering and assembling things in unconventional affordances. Creativity is basically the solution to problems in a useful way (Sonenshein, 2014). Besides these, the empirical study indicates that the behavior of the leader is an important factor in cultivating creativity (Lin et al., 2016). According to the needs of the employees, appropriate support from leaders is required for creativity (Cheung and Wong, 2011).

In order to solve a problem, employees must be self-determined, know the importance of work, be competitive, and be impactful (Zhang and Bartol, 2010; Sun et al., 2012; Amundsen and Martinsen, 2015). Psychological empowerment resulting in high-quality LMX (Schermuly and Meyer, 2016; Audenaert et al., 2017) is a consequence of LH (Yam et al., 2018), ensuring that employees show activism to solve problems. Additionally, from a theoretical perspective, as described above, the amalgamation of BVT and SIPT indicates that when a leader uses humor to violate norms, it signals what attitude is required. Employees process these signals through experience and perceive that the leader is vulnerable, permissive, and he wants to reduce hierarchical distance, acceptance of normative behavior to build high-quality LMX relations. Thus, employees attribute these high LMX relations to psychological empowerment for themselves due to the effort of the leader to establish a strong relationship. Hence, this empowerment helps in creating a positive attitude, which is creativity. Furthermore, there is almost a dead in this relationship between LH and creativity. The reason is that most researchers consider it theoretical in nature, and there is less empirical evidence that examines the impact of LH (Yam et al., 2018). However, a closer look at this prescribed relationship was studied by Pundt and Herrmann (2015). According to this study, humor is emphatically related to innovation. Similarly, the findings by Mao et al. (2017) indicated the positive impact of LH on performance, a closely related variable to creativity. Furthermore, LMX predicts creativity (Zhao et al., 2014; Wang et al., 2015; Meng et al., 2017). Similarly, LMX has a positive association with PE (Dulebohn et al., 2011; Wang et al., 2016), and PE can be used as a mediating variable (Sun et al., 2012; Amundsen and Martinsen, 2015; Fong and Snape, 2015). So, it can be suggested that LH signals to build a positive attitude *via* LMX and PE.

*H*_3:_*LH is positively linked to employee creativity* via *LMX and PE in sequence*.

### Implications for Acceptability of Norm Violation, Perceived Power, and UB

Norms are formal rules (e.g., code of conducts) or as informal perceived descriptive norms (e.g., be good with other fellows) of a particular organization (Morris et al., 2015). Norms may be resources or constraints (Morris et al., 2015). Similarly, employees do not operate in a vacuum but have fellows and leaders around them, and sketch pictures about what attitude is rewarded (Salancik and Pfeffer, 1978). Further, when leaders use humor to violate norms, employees perceive it as socially acceptable for two reasons. First, employees consider their leader as a role model (Yukl, 2010). Second, employees perceive that norm violation is not punishable. Likewise, SIPT indicates that actions of the leaders send signals to employees about what attitude is rewarded or punished in an organization. In this context, Yam et al. (2018) provided evidence about the impact of LH on the perceived acceptability of norm violation. In this study, data were collected from China and the United States. The result of this research study reveals that LH elicits positivity as wells as negativity. So, this study provides support to develop a hypothesis in this way.

*H*_4_*: LH is positively associated with the acceptability of norm violation*.

Though LH impacts norm violation, it is expected here that LH affects the increasing power of employees. Literature defined power as control over resources (money, decision making, and information). But Anderson et al. (2012) described power to influence peers. Here is a focus on this latter definition. Further, empirically, norm violators are perceived as more powerful (Stamkou et al., 2019).

As employees learn that norm violations are acceptable in their organization resulting from leader humor, they feel more powerful in doing whatever they want. The key to this postulate is the acceptability of norm violation. Indeed, people perceive more power because of accepting norm violation (Van Kleef et al., 2011) resulting from LH (Yam et al., 2018). Additionally, from a theoretical perspective, when a leader uses humor that violates norms, he gives signals or conveys some information to employees. Thus, employees process these signals through experience, and perceive acceptability of norm violation because their role model is also violating norms. Hence, it is attributed to this acceptability to perceive power because employees think they can violate norms by becoming more powerful. So, in concluding remarks, it is suggested here that acceptability of norm violation predicts perceived power as a result of LH.

*H*_5_*: LH is positively associated with perceived power and mediated acceptability of norm violation*.

Although violating norms is associated with the perception of power, it is also anticipated that LH predicts UB. It is defined as a behavior that is not up to standard or moral expectations (Dubois et al., 2015). For example, we consider it unethical if a taxi driver intentionally takes a longer path to reach a destination instead of having a shorter route to get there (Butler et al., 2016). As a leader demonstrates humor in violating norms, it leads to acceptability of norm violation, power perception, and UB in sequence. The key to this postulation is the acceptability of norm violation and power perception. Indeed, employees do some unethical activity because of power (Rees et al., 2019), resulting from the acceptability of norm violation (Van Kleef et al., 2011), predicted by LH (Yam et al., 2018). Further, with the integration of SIPT and BVT, we argue that LH gives signals to employees that norm violation is acceptable in their organization. This acceptance of violation leads to power perception. Hence, this powerful nature enhances unethical attitudes among employees because employees think that this attitude is not punishable (this link has been explained in a very detail form in the theoretical section). Empirically, literature is silent to show association of LH to UB. However, a positive relationship exists between power and UB (Lammers et al., 2010; Yap et al., 2013; Dubois et al., 2015). So, LH predicts UB through acceptability of norm violation and perceived power.

*H*_6_ *: LH is positively associated with UB of employee, mediated acceptability of norm violation, and perceived power in sequence*

### The Moderating Role of LSDH

There are justifications that effects (explained in the positive and negative path) are also controlled by a specific type of humor. Justification becomes valid after merging BVT with SIPT. Through literature, it can be predicted that LSDH helps increase its relationship with followers and increase positivity. While at the same time, LSDH perceives the followers not to violate norms and reduces negativity. It is necessary to indicate that humor (used with independent variable) is considered general (any style of humor) while deprecating humor is a specific style of humor. Humor has different styles, such as self-deprecating, self-defeating, aggressive, and self-enhancing (Martin et al., 2003). Here is a focus on self-deprecating humor as suggested by Yam et al. (2018) for future researchers to check whether it plays a role of moderating variable or not. Self-deprecating is self-joking (Martin et al., 2003). But how it can be used as a moderating variable will be discussed in the next paragraph.

This research argues that norm violations can be reduced when leaders use a deprecating style of humor. The reason is that when leaders use deprecation humor coupled with their sense of humor, they violate norms but not at a severe level, signals not to violate the social norms of civility. Thus followers perceive it as being respectful to others or at standards of morality during social interaction. As employees perceive their leader as a role model (Yukl, 2010), they have no acceptance of violating norms. Memili et al. (2013) suggested that cohesiveness, a form of self-deprecating humor, reduces conflicts because organizational bodies move in one direction as they know their role. Moreover, Martin et al. (2003) considered that self-deprecating humor as a nonhostile form. The nonhostile nature of self-deprecating humor decelerates norm violation when leaders use humor because of its self-joking nature based on norm violation but not at a severe level. Based on these arguments, it is postulated that LH signals nonviolating norms behavior in the presence of self-deprecating humor.

*H*_7_*: The indirect effect of LH on UB*, via *acceptability of norm violation and power in sequence, is moderated by LSDH such that the indirect effect is weaker when LSDH is high, but stronger when it is low*.

Similarly, leaders with strong humor sense often use self-deprecating humor to develop strong relations with employees and create positive consequences. Leaders use self-joking in self-deprecating humor to amuse followers and impose themselves for relationship orientation (Martin et al., 2003). According to this research, self-deprecating humor is nonhostile, averring self and followers to increase cohesiveness or interpersonal relations. Moreover, this research specifies that self-deprecating humor is connected with cheerful, extroversion, relationship-oriented, self-esteem, positive moods, etc. It means LH in the presence of self-deprecation strengthens the follower relationship. Stewart (2011) indicated equal relationships are developed between audience and speaker who use self-deprecating humor. Here a question arises that how LSDH increases the relationship between followers and leaders (Stewart, 2011). According to this research, self-deprecating humor demises the status distance between followers and leaders. Kim et al. (2016) stated that public relations strategists often use self-deprecating humor.

Moreover, it is seen that the self-deprecating humor used by Obama during his election campaign in 2008 impacted in engaging followers and enhancing the relationship with the followers (Stewart, 2011). Another research conducted by Kim et al. (2016) highlighted that Alibaba (a large Chinese company) uses a self-deprecating humor strategy during crises on social media.

*H*_8_*: The indirect effect of LH on employee creativity*, via *LMX and PE in sequence, is moderated by the self-deprecating humor of the leader such that the indirect effect is stronger when the self-deprecating humor of the leader is high, but weaker when the self-deprecating humor of the leader is low*.

**Figure 1:**
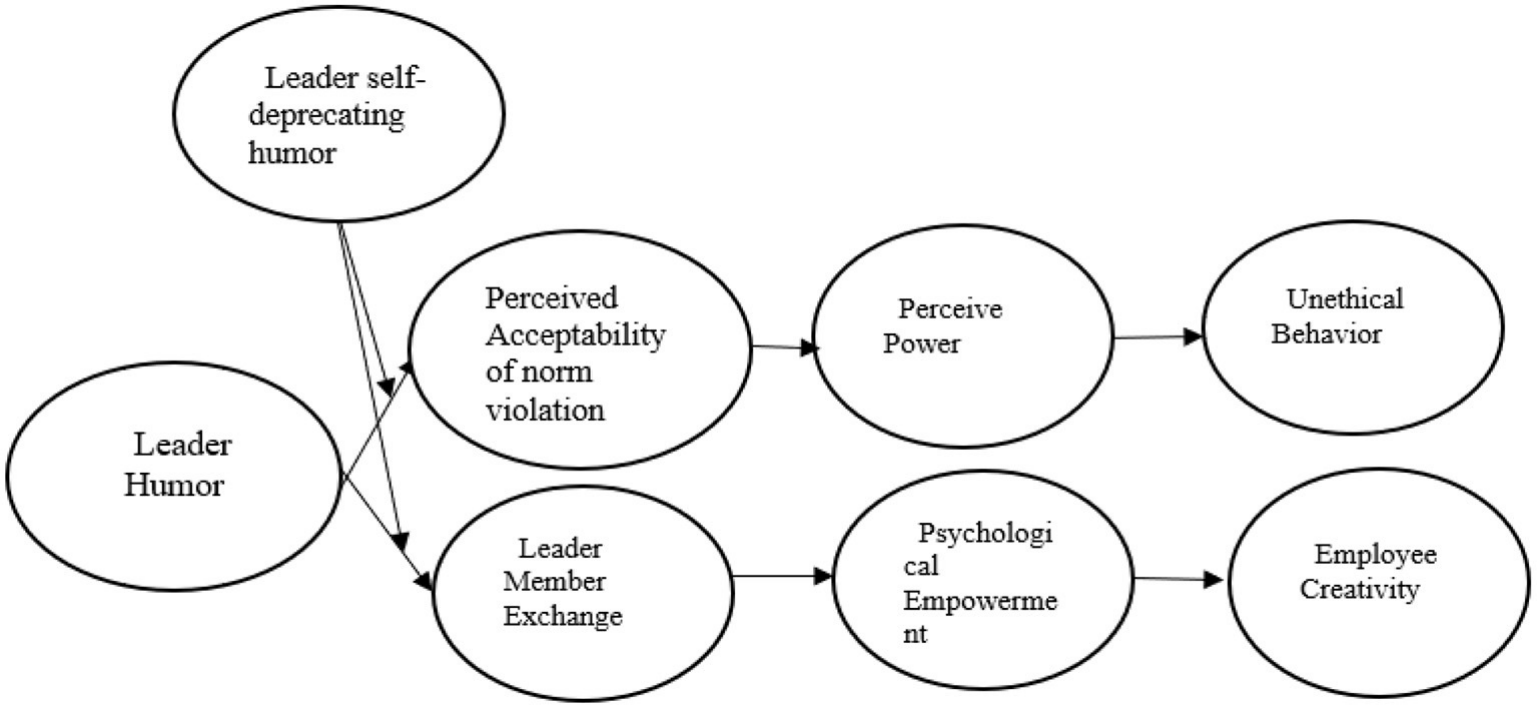
Framework of current study. In this figure, perceived acceptability of norm violation and perceived power are sequence mediation between leader humor (LH) and unethical behavior (UB) (curse path), while LMX and PE are also sequenced mediation between LH and employee creativity (blessing path). Moreover, the self-deprecating humor of the leader (LSDH) plays the role of a moderating variable.

Based on these arguments, Figure 1 depicts the theoretical framework developed for this research.

## Research Methodology

### Sample and Procedure

Data were collected from the top ten software houses listed with Pakistan Software Export Board and using the quota sampling technique. A nonprobabilistic technique of gathering data from individuals representing the population by first fixing the quota and then employing convenience sampling. Researchers use this sampling technique for two reasons: Infinite or unknown population and inaccessibility (Yang and Banamah, 2014), and the latter was the reason for selecting this technique in the current study. Moreover, market and social sciences research heavily rely on this sampling technique as a default technique (Ochoa and Porcar, 2018). The sample size is 630 for this study, as suggested by Hair et al. (2011), and for a 70% expected response rate, 900 questionnaires were distributed. Moreover, the unit of analysis is an individual (employee), comprising each quota of 90 respondents for each software house. The software houses from which the data were collected are as follows: Netsol Technologies, System Limited, I2C Pakistan, S and P Global (Pvt) Ltd, TRG Pakistan-Afiniti, LMK Resources Pakistan (Pvt) Limited, Mentor Graphics Pakistan (Pvt) Limited, Teradata Global Consulting Pakistan (Pvt) Limited, Ovex Technologies Pakistan (Pvt) Limited, and Medical Transaction Billing Co. (Pvt) Limited. According to Pakistan Software Export Board, Netsol Technologies software house has the highest sale of over $20 million, followed by Teradata Global Consulting Pakistan (Pvt) Limited, TRG Pakistan-Afinti, System Limited, S&P Global (Pvt) Ltd, which have exports from $10 to 20 million, and then I2C Pakistan, LMK Resources Pakistan (Pvt) Limited, and Mentor Graphics Pakistan (Pvt) Limited have exports of $ 5 up to 10 million in a year (Table 1).

**Table 1:** Demographic characteristics of the respondents.

**Category**	**Frequency**	**Percentage**
**Gender**		
Female	136	21.6
male	494	78.4
Total	630	100.0
**Age**		
20 years_less than 30	496	78.7
30 years_less than 40	118	18.7
40 years_less than 50	16	2.5
Total	630	100.0
**Experience**		
5 years and less	450	71.4
5 years_less than 10	154	24.4
10 years_less than 20	26	4.1
Total	630	100.0

Data were collected from software houses for multiple reasons. First, software engineers have a very dull life, and they need more humor in their work settings than the employees working in other industries or companies. Second, software houses have to bear a lot of costs on implementing different structural policies. Third, software houses work on various creative projects, custom software solutions, and require thinking “out of the box.” These companies demand more creative work from their employees because every project requires creativity rather than other companies, which mostly follow similar processes for developing their products. So, our developed framework for this research has congruence with software houses. More importantly, the software industry has enormous potential in creating jobs and resolving the economic issue nowadays. The development of strategies and plans to resolve problems and enhance the performance of such a sector is fruitful in a country like Pakistan, which faces budget deficit issues from foreign exchange.

Among the respondents, 136 were women, while 494 were men, and most of them had an experience from 5 to 10 years (450), while some had an experience from 5 to 10 years (154) and very few were with experience from 10 to 20 years, 26 in numbers. Moreover, 496 software engineers were aged from 20 to 30 years, while between 30 and 40 and 40 and 50 years, they were 118 and 16 in numbers, respectively. The primary data were collected from these software engineers using a 7-point Likert scale, and 630 numbers of the questionnaires were appropriate for use after screening and cleaning the data.

Given the cross-sectional nature of the study, the findings might be prone to what is known as common method bias because of common method variance (CMV) (Podsakoff et al., 2003; Spector and Brannick, 2009). There are two approaches to control CMV, ex-ante and ex-post. The present study first adopted the ex-ante approach during the research design stage. The respondents, ensuring their secrecy and identity, were emphasized that the answers should be honest without considering them right or wrong (Podsakoff et al., 2003). Moreover, the order of items of all the constructs, including independent, dependent, and moderator variables, was mixed to avoid CMV-biased pattern of responses in intellectually establishing the “required” correlation (Murray et al., 2005). In addition, the complexity of the current theoretical model (comprising moderator and mediators) helped reduce in constructing a cognitive map of interaction and nonlinear effects (Harrison et al., 1996). Then the study applied an ex-post approach to determine the biasness through statistical techniques. In this regard, a *post hoc* test known as Harman's single factor was applied without rotating the factor (Chang et al., 2010). The single factor contributed 28% variance, which was less than the customary threshold of 50%. Moreover, the cumulated variance of all factors was 68%, strengthening the inference. However, Podsakoff et al. (2003) illustrated that this test is insensitive because it is improbable that a single factor would fit the entire data, and, furthermore, no worthwhile threshold is available. Consequently, confirmatory factor analysis of the single factor was run in order to examine the data fitness to the hypothesized model (Malhotra et al., 2006). The data were a poor fit for the single factor: [Degree of freedom (DF) = 10.651, normed fit index (NFI) = 0.386, incremental fit index (IFI) = 0.212, Tucker-Lewis index (TLI) = 0.329, goodness of fit index (GFI) = 0.321, adjusted goodness of fit index (AGFI) = 0.291, root mean square error of approximation (RMSEA) = 0.189, and root mean square residual (RMR) = 0.243]. It implied the absence of CMV. Lastly, common latent factor (CLF) was used to assess biasness. The deviations <25% of the standardized regression weights of the model in the presence and absence of CLF proved that CMV is not a concern in this study (Williams et al., 1989).

### Measures

The already developed scales have been used in this study to capture the response of the participants. All the items were listed on the Likert scale [1 is “strongly disagree” and 7 is “strongly agree”].

The measures for LH were adopted from Yam et al. (2018). The reported Cronbach's alpha value is 0.96. The number of items is seven on this scale. The sample item is “My leader says things in such a way as to make people laugh.” Van Kleef et al. (2011) developed the “*Perceived Acceptability of norm violation”* scale, and used it for measuring norm violation acceptance. In this study, the same 7-item scale was used with Cronbach's alpha value is 0.77. The sample item includes, “To what extent you think it is acceptable for a person in the organizations to be *immoral.”*The *LMX* scale was adopted from Yam et al. (2018), having Cronbach's alpha value of 0.96. This scale has eight items, and the sample item for this scale includes, “I usually know where I stand with my leader.”

Spreitzer (1995) developed twelve item-*Psychological Empowerment* scale, and it has reported Cronbach's alpha value of 0.72. An example item includes “The work I do is very important to me.” Similarly, four item-scale developed by Yang et al. (2019) was used to assess employee creativity responded by supervisors. The reported Cronbach's alpha value was 0.87. The sample item includes, “I suggest new ways to achieve goals or objectives.” The eight item-scale developed by Anderson et al. (2012) was used for measuring perceived power, assessed through subordinates. Cronbach's alpha value was 0.81 with the sample item, “I can get others to listen to what I say.”

Likewise, a scale from Jacobs et al. (2013) was used for measuring UB. Cronbach's alpha value was 0.88. An example item of this scale includes, “I purposely wasted company materials/supplies.” The scale developed by Martin et al. (2003) was used to measure self-deprecating humor, assessed through subordinates. Cronbach's alpha value was 0.80. The sample item includes, “My leader does not have to work very hard at making other employees laugh—My leader seems to be a naturally humorous person,”

## Data Analysis and Results

Structural equation modeling (SEM) was used to analyze data in the current study. The SEM is a popular technique in behavior and social sciences equipped to handle measurement error, multiple equation models, and multiple measures for concepts (Bollen and Noble, 2011). The SEM is a statistical approach comprising two component model: one is the measurement model, and the second is the structural model. In other words, confirmatory factor analysis (CFA) is the measurement model, while the structural model is the multiple regression mode l (Hoyle, 1995). AMOS 21 has been used for analysis purposes, designed to implement a general approach to data analysis (Arbuckle, 2011).

### Confirmatory Factor Analysis (Measurement Model)

The researchers require CFA before testing hypotheses to prespecify all the aspects of the model (Lam et al., 2016). Basically, CFA demonstrates whether factors indicate a good fit to the data (model fit) (Lin et al., 2018), and checks whether reliability and validity score is up to the mark (Troester and Quaquebeke, 2020). The values of model fit indices for the current study, such as root mean square error of approximation (RMSEA) 0.62 <0.8, minimum discrepancy per degree of freedom (CMIN/ DF) = 3.43, comparative fit index (CFI) = 0.927 ≥ 0.90, confirm that structural model is a good fit to data (Lubatkin et al., 2006; Kenny et al., 2014; Thaichon et al., 2014; Caputo et al., 2019; Briggs et al., 2020).

**Figure 2:**
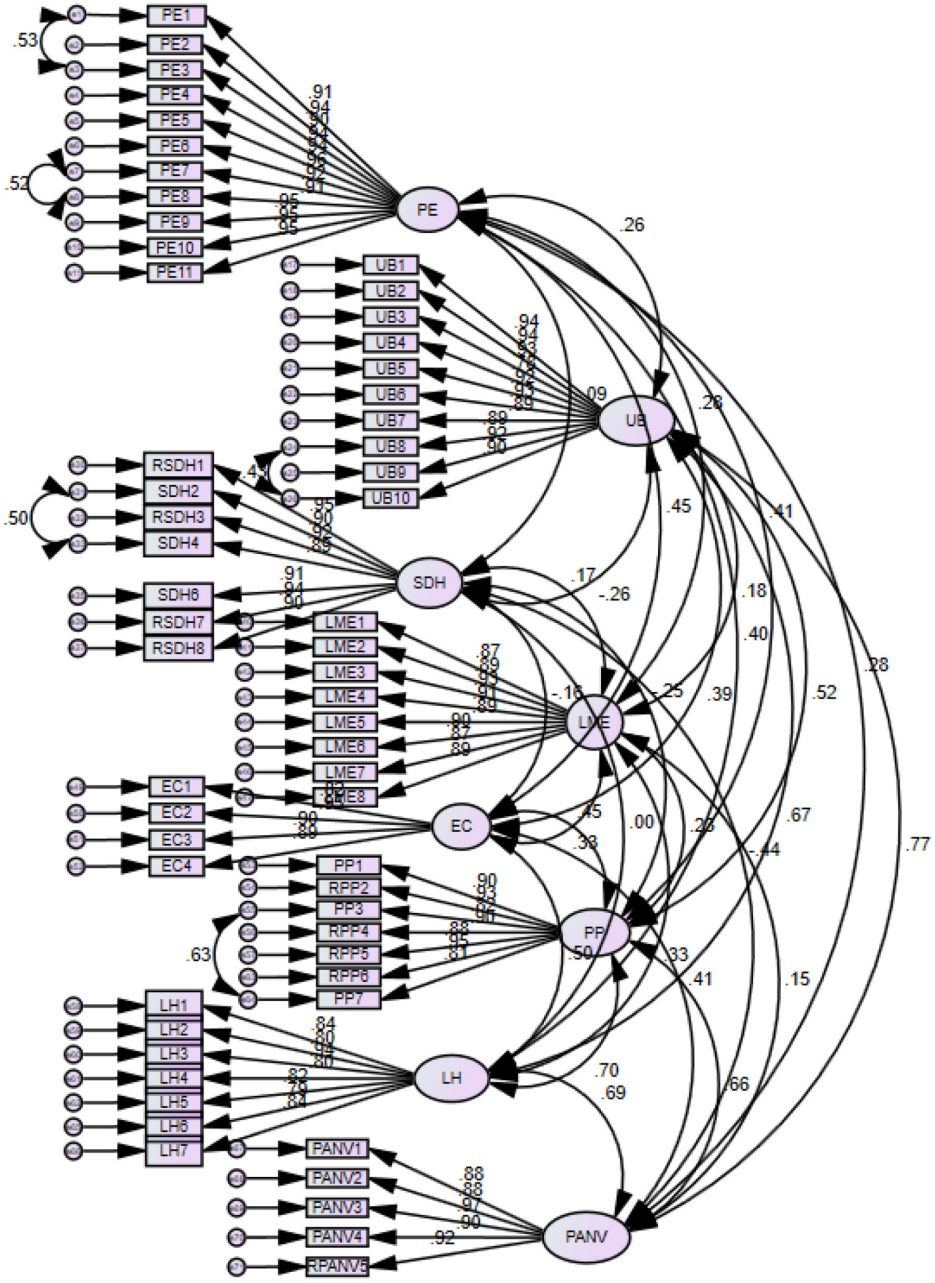
Confirmatory factor analysis.

Figure 2 represents the graphical representation of CFA, double-headed arrows indicate the covariance between variables, values on each variable are values of variance, (scattering of data around mean or simply known as R-square). The values on the arrowheads (arrows from variables to items) represent the intercept values, and values on these arrows represent factor loading values. Moreover, residuals were correlated to reduce redundancy because two items had the same meaning, and values on arrowheads (arrows from residual to items) are intercept values. The intercept values are zero due to standardized estimates in Figure 2.

Furthermore, reliability and validity are also part of the measurement model used to test the measurement model. *Reliability* can be measured by Cronbach's alpha, and its threshold value is above 0.70 for all scales (Mulki and Lassk, 2019). It is important to note that Cronbach's alpha is used when researchers conduct exploratory factor analysis, while composite reliability (CR) is preferred when researchers perform CFA to determine internal consistency. In this research, both CR and alpha values were assessed, and it can be seen from Table 2 that all values are above 0.70, indicating that the values are within the range (Lee et al., 2020).

**Table 2:** Validity and reliability tests.

**Variable**	**Factors**	**Loadings**	**CR**	**Alpha**	**AVE**
Psychological empowerment	PE1	0.906	0.987	0.987	0.870
	PE2	0.937			
	PE3	0.900			
	PE4	0.935			
	PE5	0.940			
	PE6	0.962			
	PE7	0.918			
	PE8	0.911			
	PE9	0.949			
	PE10	0.952			
	PE11	0.946			
Unethical behavior (UB)	UB1	0.942	0.979	0.981	0.823
	UB2	0.942			
	UB3	0.935			
	UB4	0.783			
	UB5	0.921			
	UB6	0.932			
	UB7	0.890			
	UB8	0.890			
	UB9	0.924			
	UB10	0.904			
Self-deprecating humor of the leader	RSDH1	0.946	0.973	0.974	0.836
	SDH2	0.897			
	RSDH3	0.923			
	SDH4	0.889			
	SDH6	0.911			
	RSDH7	0.937			
	RSDH8	0.897			
Leader–Member Exchange (LMX)	LMX1	0.868	0.969	0.954	0.798
	LMX2	0.890			
	LMX3	0.926			
	LMX4	0.913			
	LMX5	0.889			
	LMX6	0.898			
	LMX7	0.870			
	LMX8	0.892			
Employee creativity (EC)	EC1	0.820	0.939	0.938	0.793
	EC2	0.949			
	EC3	0.900			
	EC4	0.890			
Perceived power	PP1	0.901	0.962	0.964	0.786
	RPP2	0.932			
	PP3	0.818			
	RPP4	0.903			
	RPP5	0.877			
	RPP6	0.952			
	PP7	0.812			
Leader humor (LH)	LH1	0.836	0.941	0.940	0.696
	LH2	0.803			
	LH3	0.940			
	LH4	0.802			
	LH5	0.823			
	LH6	0.787			
	LH7	0.843			
Perceived acceptability of norm violation (PANV)	PANV1	0.882	0.961	0.960	0.832
	PANV2	0.884			
	PANV3	0.968			
	PANV4	0.902			
	RPANV5	0.921			

*Similarly, convergent validity is the most common form of validity used to test measurement models. It is* measured by average variance extracted (AVE > 0.50) or standardized factor loadings (Lee et al., 2020). Standardized factor loading value should be greater than 0.50, but if values are <0.50, then items are not valuable and should be deleted (Joseph and Chin, 2019). So, due to low scores, the following items were deleted from psychological empowerment, perceived power, variable, UB, and LSDH, respectively: “I have significant influence over what happens in my department.” “I think I have a great deal of power.” “I complained about insignificant things at work.” “My leader usually doesn't like to tell jokes or amuse employees (R).” Similarly, Table 2 indicates that AVE values are above the threshold value of 0.5, establishing the convergent validity (Cheung and Wang, 2017).

### Structural Model

**Figure 3:**
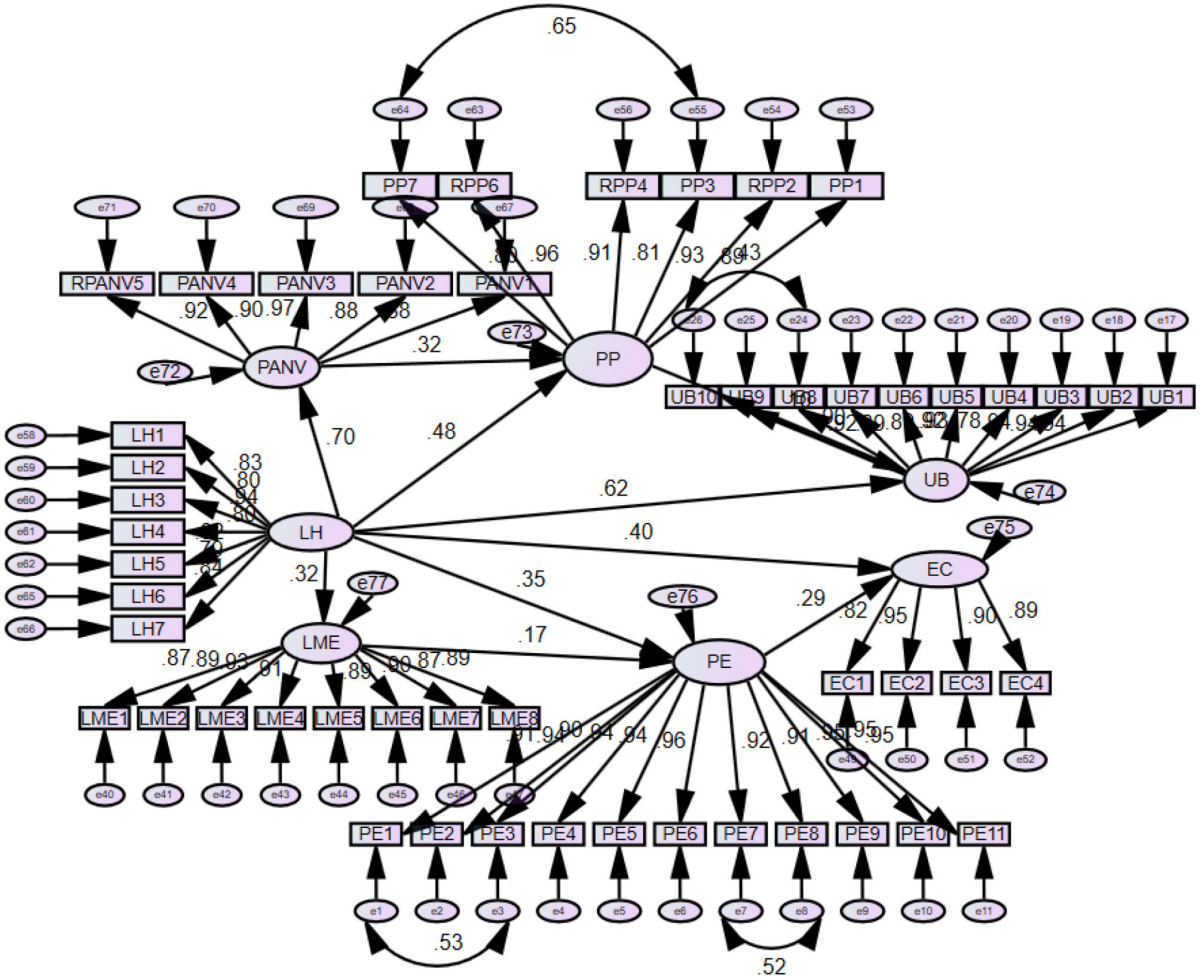
Structural model.

After CFA, the next is to determine the general path coefficients to assess whether there is a negative or positive direction, level of correlations, and significance between variables. Analysis of moment structures (AMOS) was undertaken for path coefficients of the current study (Darvishmotevali et al., 2020). Figure 3 is a graphical representation of the structural model drawn in AMOS software.

From this graphical representation, values have been extracted in Table 3, representing path coefficients with critical ratio (CR = Estimate/SE) and *p*-values. Suppose an estimate is represented by a negative value, the exogenous and endogenous estimates have a negative direction between them, and if the estimate has no sign, then by default, it represents the positive sign, which means exogenous and endogenous estimates have a positive direction between them (Caputo et al., 2019; Chang et al., 2020; Zhang et al., 2020).

**Table 3:** Structural model summarized (direct effects).

**Paths**	**Estimates**	**SE**	**CR**	**P**
LMX < −−−LH(H1)	0.316	0.040	7.909	***
PANV < −−−LH (H4)	0.857	0.047	18.336	***
PP < −−−PANV	0.240	0.032	7.578	***
PE < −−−LMX	0.223	0.052	4.300	***
PP < −−−LH	0.431	0.041	10.624	***
PE < −−−LH	0.455	0.053	8.620	***
UB < −−−PP	0.148	0.069	2.145	0.032
EC < −−−PE	0.179	0.024	7.488	***
UB < −−−LH	0.858	0.067	12.719	***
EC < −−−LH	0.313	0.033	9.640	***

***p < 0.001.

It can be observed from Table 3 that all values of estimates are positive and *p*-values are below 0.01, indicating a positive and highly significant relationship between variables except PE to employee creativity path, which have a positive relationship but not highly significant.

Table 4 shows the findings of mediation and sequence mediation hypotheses. This table shows that Hypothesis 2 is supported, given that LH is positively predicted by PE *via* LMX (B = 0.071, CI of 95% = 0.039–0.117). Hypothesis 3 supports that LH positively predicted employee creativity via LMX and PE in sequence (B = 0.094, CI of 95%= 0.075–0.119). Likewise, Hypothesis 5 is accepted, indicating that LH positively predicted perceived power via perceived acceptability of norm violation (B = 0.206, CI of 95% = 0.150–0.265).

**Table 4:** Summarized results of mediation and sequential mediation hypotheses.

**Hypotheses**	**Estimates**	**LLCI**	**ULCI**	**Results**
Leader humor (LH) is positively linked with psychological empowerment (PE) *via* leader-member exchange (LMX) (H2)	0.071	0.039	0.117	Supported
LH is positively linked with employee creativity *via* LMX and PE in sequence (H3)	0.094	0.075	0.119	Supported
LH is positively associated with perceived power, mediated acceptability of norm violation (H5)	0.206	0.150	0.265	Supported
LH is positively associated with employee unethical behavior (UB), mediated acceptability of norm violation and perceived power in sequence (H6)	0.094	0.003	0.191	Supported

*From the values of LLCI and ULCI, it can be seen that zero does not exist between their intervals, providing support for the hypotheses*.

Similarly, Hypothesis 7 states that LH positively predicts UB of employee via perceived acceptability of norm violation and perceived power in sequence (B = 0.094, CI of 95% = 0.003–0.191), and this hypothesis is also accepted. Since direct and indirect paths are significant, we infer that partial mediations exist among the variables.

Furthermore, the interaction effects of LH and LSDH on PANV were also examined with the help of PROCESS macro 3.4 (Hayes, 2017), as shown in Table 5.

**Table 5:** Summarized results of moderating variable.

**PATHS**	**Coeff**.	**T**	**P**	**LLCI**	**ULCI**
LH > PANV	0.8482	21.9370	0.0000	0.7722	0.9241
LH > LMX	0.4500	13.3812	0.0000	0.4075	0.5477
LSDH > PANV	−0.4587	−17.3667	0.0000	−0.5105	−0.4068
LSDH > LMX	0.1353	−2.5978	0.0096	−0.1112	−0.0155
Interaction_1 > PANV	−0.0726	−3,2727	0.0011	−0.1161	−0.290
Interaction_2 > LMX	0.1386	7.4764	0.0000	0.1129	0.1933

Interaction, leader's self-deprecating humor; LH, leader's self-deprecating humor (LSDH); *LH; PANV, perceived acceptability of norm violation.

**Figure 4:**
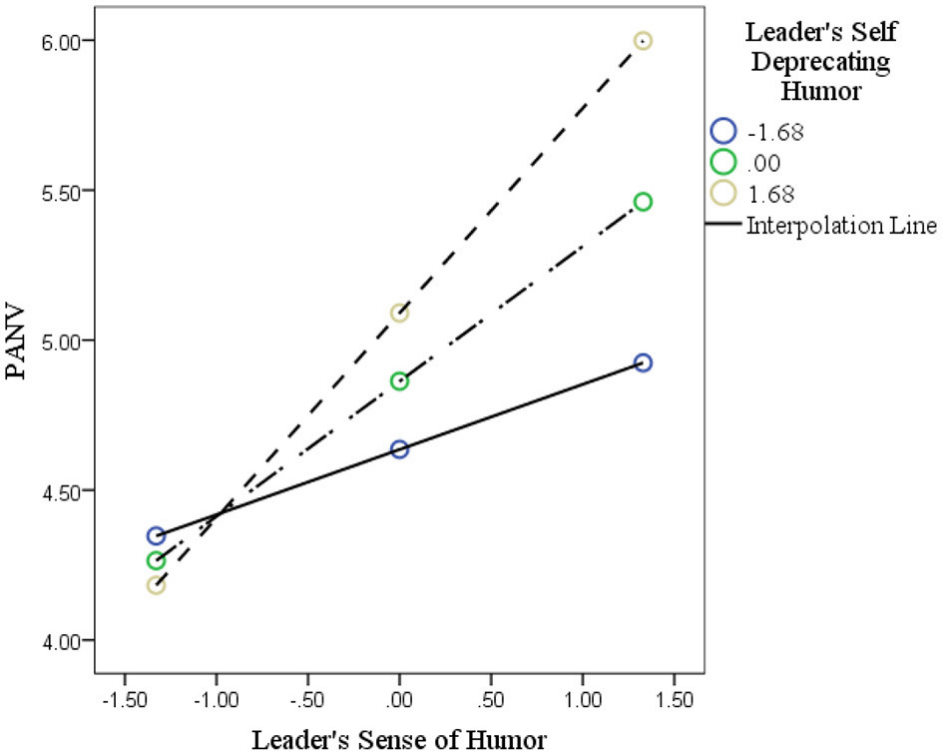
The interaction of leader's self-deprecating humor and leader's humor on PANV.

In Step 1, the LSDH is negatively associated (−0.4587, *p* < 0.05), while LSDH (0.8482, *p* < 0.05) was positively and significantly linked with PANV. In Step 2, the interaction of the LSDH and the LH (B= −0.0726, *p* < 0.05) was negatively linked with PANV, providing support for this hypothesis. The negative sign of the beta value of interaction indicates that the product of LH and LSDH reduces the effect of the LH variable. The interaction effects have been plotted in Figure 4. The interacting effect is not visible in the first quadrant but would be visible if extrapolated to the second quadrant due to the negative relationship.

**Figure 5:**
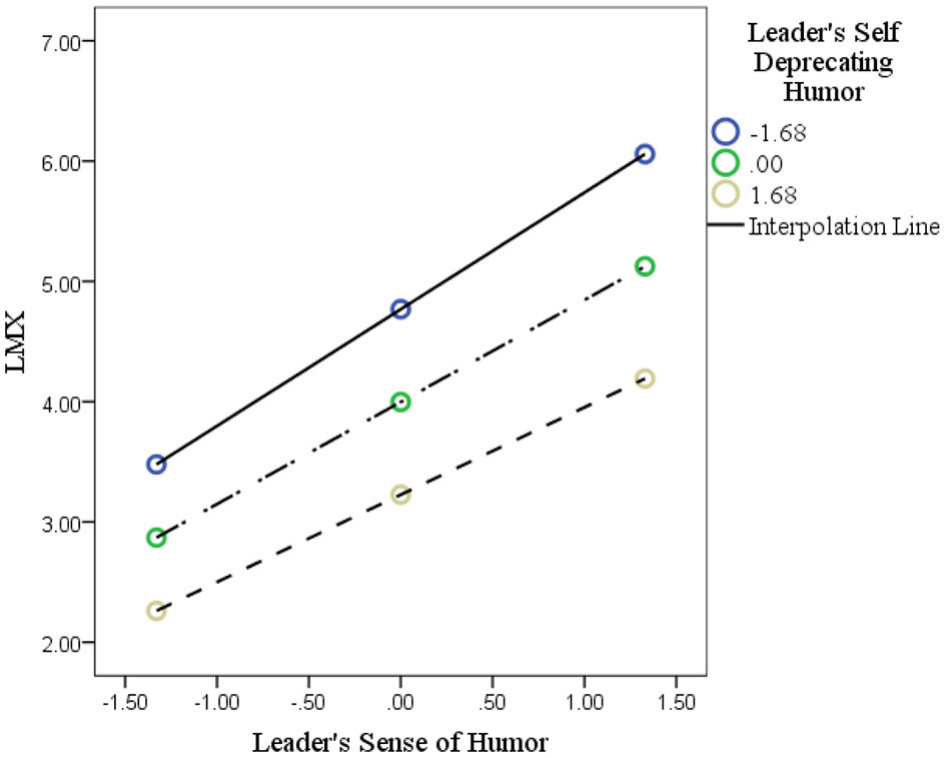
The interaction of leader's self-deprecating humor and leader's humor on LMX.

Similarly, Hypothesis 8 was tested in the same way by testing the interaction impact of LSDH and LH on LMX. In Step 1, LH (B = 0.4500, *p* < 0.01) and LSDH (B = 0.1353, *p* < 0.05) were positively associated with LMX. In Step 2, the interaction effect of LH and LSDH was positively associated with LMX (0.1386, *p* < 0.01), supporting this hypothesis. The interaction effect has been plotted in Figure 5.

## General Discussion

**Figure 6:**
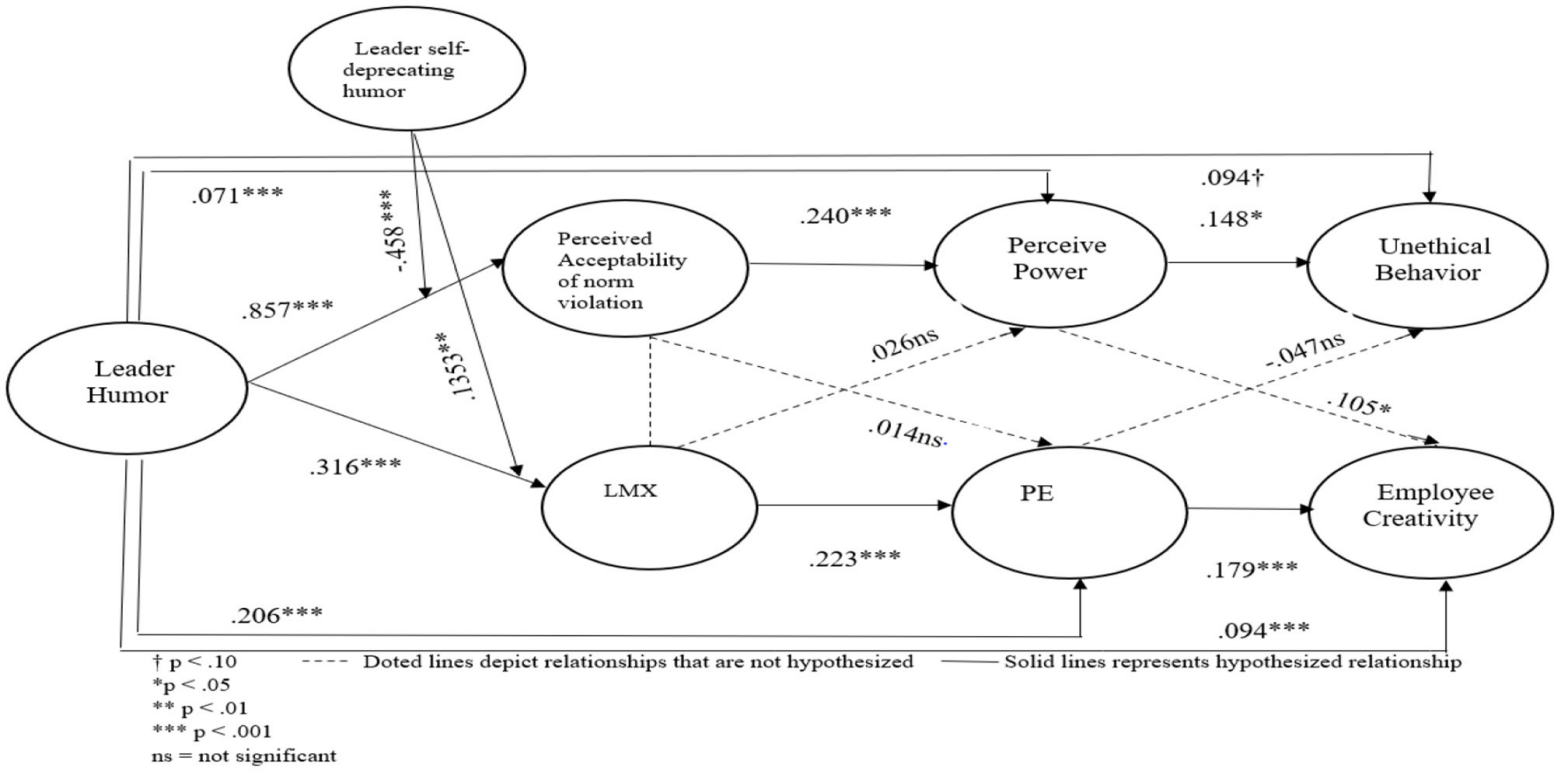
Summarized results of paths models and hypotheses of current study.

The field study provided consistent support for all hypotheses. The results revealed that LH is a double-edged sword. On the one hand, it signals the acceptability of norm violation which, in turn, leads to UB *via* perceived power. On the other hand, it is associated positively with employee creativity *via* leader-member exchange and psychological empowerment in sequence. Further, it was also demonstrated that LSDH enhances positivity and reduces negativity, emerging from LH. In simple words, a humorous leader who tends to use self-deprecating humor is more likely to discourage UB and promote creativity. The summary of findings of the current research can be seen in Figure 6. In this figure, dotted lines depict relationships that are not hypothesized, while solid lines depict the hypothesized relationship. Further, theoretical and practical implications and limitations and suggestions for future directions have also been discussed in the subsequent subsections.

### Theoretical Implications

This study makes many theoretical contributions. First, introducing BVT in explaining the research framework would help researchers trace the effects of humor more systematically in organizational settings. More importantly, BVT was developed initially to explain whether the act is humorous or not. By integrating with SIPT, we enrich BVT by studying the effects and processes of LH. Moreover, by the integration of theories in formulating the framework, the current research enriches the literature in a sense that LH not only has a direct impact on employee creativity and UB but also with a specific indirect effect. Further, researchers emphasize more empirical research on humor, especially on LH (Pundt, 2015; Robert et al., 2015; Yam et al., 2018; Kong et al., 2019). In this regard, the current study is unique in applying BVT with SIPT in the workplace to develop and empirically study a comprehensive framework. Moreover, BVT assumes humor as a benign norm violation while organizations have norms, which are practices. This research provides an impetus to study BVT with other theories in other workplace environments, such as tourism, fashion, and entertainment, where norms are not practices. Here, violations are considered fine, and employees often use humor. The LH may not convey norm violation in such industries rather than norm adherence.

Furthermore, BVT states that breaking rules are beneficial (McGraw and Warren, 2010). Briefly speaking, it is fine to violate norms having nonthreatening nature, or be it benign, in other words. While LH sends a powerful message that violations are acceptable in the organization, it implies that with the use of humor, the leader is also violating norms in the sense of breaking normal hierarchical distance (reducing distance between employees and himself). When employees observe that their role model is also violating the norms, they perceive it acceptable to break some existing working rules, which allow employees *to identify new ideas*; hence, leading to creativity. So, in this way, this study has extended the literature related to BVT, which was previously just limited to identify humorous and nonhumorous acts. Likewise, the existing literature seems to have very limited evidence of the repercussion of LH using BVT. As stated in the paragraph above, LH conveys the acceptance message for violating norms to employees but has different implications of enhancing UB rather than enhancing creativity. Since BVT defines humor as benign norm violations, and leader also violates norms when using humor, leading to the acceptability of norm violation. When norm violators perceive themselves as powerful (Stamkou et al., 2019), they do more corruption because power is corrupt in itself (Dubois et al., 2015). Hence, the development of this negative link (UB) would enable researchers to advance BVT.

Second, research with multiple paths and consequences is scarce, and researchers encourage future researchers to study this avenue (Kong et al., 2019; Karakowsky et al., 2020). This study broadens the literature by uniquely unfolding the impact of LH on the dual behavior of the followers. It assumes LH as a double-edge sword with two sequential mediators on both the edges, along with one moderator, grounded on the integration of BVT and SIPT. Third, this study is first to investigate how to control the negativity of LH to make it more productive. Previous literature indicates that research focuses on either positivity or negativity, ensuing LH (Cooper et al., 2018; Yam et al., 2018; Kong et al., 2019). This research advances the literature by controlling the negativity of LH while enhancing its positivity. For this, LSDH has been used as a moderating variable in this current study. Fourth, the existing research is limited to modeling frameworks limited to very little understanding of LH (Goswami et al., 2016; Karakowsky et al., 2020). Furthermore, Yam et al. (2018) suggested additional research while using BVT and SIPT on LH. This study expands the literature by providing a more comprehensive rationale of how LH shapes the behavior of the followers. In this way, this research provides a deep understanding of how to examine humor impacts in organizational settings.

Fifth, previous studies focused on one mediation of blessing (Hu and Luo, 2020; Karakowsky et al., 2020). The researchers have missed the other elements (Gkorezis et al., 2014; Wijewardena et al., 2017; Bitterly and Schweitzer, 2019; Hu and Luo, 2020). In this context, the current research has incorporated sequential mediation of leadership relations to advance the literature. For this, two sequential mediations, norm violation and perceived power, with UB as the dependent variable was used in the negative path and two sequential mediations, LMX and PE, with the creativity used in a positive path. At the same time, LH has been used as the independent variable for both studies. Sixth, humor is a broader concept and, therefore, unable to provide a sound understanding of consequences. That is why a narrow facet variable, LSDH, has been used as a moderating variable to understand the consequence of humor better. In this way, this study advances the literature by analyzing the narrow facet variables with broader concepts.

### Practical Implications

Different practical implications are associated with this study. First, the current research would make the managers or leaders realize that their actions can be seen as cues that affect the performance of their followers both positively or negatively. Mahsud et al. (2010) indicated that followers perceive their leader as a role model. So, leaders must be the role models and track their actions to ensure what type of humor is appropriate for different situations. Second, the study suggests that a leader can increase benefits (employee creativity) and reduce curses (UB) through the proper use of humor. As it is a low-cost strategy compared to other structural policies, it may vastly benefit the managers. Third, according to BVT, it is imperative and beneficial to break norms. Hence, LH sends an implicit message that breaking the rules is fine. Thus, employees understand that violating some existing norms is forgivable in the workplace, and that it is safe to break some working rules. So, the current study corroborates that this environment at the workplace can lead to “think out of the box,” thereby enhancing creativity. In other words, the atmosphere of an organization plays a vital role in human practices and innovations. Therefore, organizations can create a culture of condonation through LH on the failure of the employees to attempt new ideas. Indeed, the use of humor by leaders facilitates in making the organization to be innovative.

Fourth, in general, humor is considered a motivational factor in enhancing performance; however, our research indicates a potential risk associated with humor. It should be clear here that we are not suggesting that the leaders should stop the display of humor in the work setting. Indeed, our framework suggests leaders resorting to humor, but it also recommends the frequent use of self-deprecating humor to enhance the performance of their organizations. Fifth, our study also reveals that LH significantly impacts UB when LSDH is not considered. Another way to make LH the most effective with self-deprecating, organizations may train their employees to espouse organizational norms. This can be done by realizing organizational identification to employees. When they think that they are an integral part of their organization, they may behave with congruence for the benefit of an organization. So, with identification, LH can be a more powerful tool in enhancing performance and creativity of the employees.

Sixth, current research indicates that LH positively impacts organizational performance in enhancing employee creativity (an integral part of making an organization more profitable and imperative for staying in business). Previously, successful leaders often use humor to motivate their employees, garner support, and even create reminiscences. For example, an angry protest smashed an egg on Arnold Schwarzenegger, the ex-governor of California. In an interview with media, the Governor responded and said, “this guy owes me bacon now.” This response of the Governor got succor in converting hatters into supporters. Indeed, practitioners often praised LH to enhance satisfaction and performance of employees (Katz, 1996). Given its perceived benefits, business and political leaders often have appointed trainers for making their leadership more effective (Yam et al., 2018). Seventh, very interestingly, it can be seen from the findings that employees who feel powerful in their actions tend to engage in more creative behavior. Thus, organizations should formulate a policy for making employees more powerful in their actions so that the organizations may perform better.

## Conclusion

In this research study, BVT and SIPT were integrated and found that LH is a double-edged sword, having a blessing and a curse for an organization. The results indicate that it leads to increased employee creativity (blessing path) and enhanced UB (curse path). Further, LSDH increases employee creativity and reduces UB of employee stemming from LH by moderating the indirect effects for both paths. We hope that this study can spark more research on LH in organizational settings. Although, this research study sheds light on the consequences of LH, however, many questions discussed in the next section need to be addressed in future studies.

## Limitations and Recommendations for Future Research

First, in this study, only the LSDH was tested as a moderating variable and it is recommended that future researchers explore other styles of LH as a boundary condition. For example, future researchers might explore whether LSDH enhances the positivity or negativity that emerges from LH. With reference to the style of the humor, Martin et al. (2003) had provided details. Moreover, a complete leadership process not only involves the trait of the leader but also involves the trait of the follower. So, the trait of the follower can be measured as a potential moderator in the relationship between a LH and follower outcomes.

It is also important that future researchers might try to answer these questions. Does LH possess the same effects for male vs. female followers? Has LH the same effects for male vs. female leaders? In other words, the moderating role of male and female leaders should be examined in the future research to identify whether both male and female leaders moderate the effects of LH in the same way or in a different way. In fact, it is a potential avenue for future researchers to consider a moderating role of gender (male vs. female leaders and also male vs. female followers) on the effects of LH because only one paper was found on the topic, which provides little knowledge. It states that female leaders are rewarded more for relationship building than male leaders when using positive humor at the workplace (Decker and Rotondo, 2001).

Furthermore, although many organizations and industries treat norm violation as a negative behavior while some industries like tourism, entertainment explicitly violate norms. So, it indicates that the conflict of industries concerning norm violation, and in this sense, industry type or organizations can be treated as a moderator in future research. Although an effort was put to develop a comprehensive framework for LH in the current study, there is still a need to research LH in the future. For example, future researchers might consider the other outcomes of LH because research on LH is very limited in an organizational context (Karakowsky et al., 2020).

In this study, SIPT with BVT was used to develop hypotheses. According to SIPT, employees receive signals from the social environment and after processing, they develop behavior, favorable or unfavorable for the organization. This signaling process is patchy without the capacity for accurate interpretation by the receiver (Connelly et al., 2010). So, future researchers might endeavor to examine the factors that can influence receivers to interpret the message delivered in the sense of humor, accurately. With the trait of followers, future researchers can also strive to identify the characteristics of the leader, which can influence the effectiveness of humor as a signaling tool. Future researchers might use signaling theory to research LH, a very similar theory to SIPT. In LH and follower context, this theory provides that LH is perceived as a cue (Cann et al., 2016).

Data were collected limited from the software houses, the IT industries, located in Pakistan. Therefore, it is recommended for future research to collect data from other industries, such as the textile industry or construction industry to generalize the findings. Further, it is also suggested to collect data from two industries and compare their results. Moreover, data were collected from employees in this study; however, it is also advised to collect data from leaders and employees. Besides, cross-sectional data were collected due to the time restrictions and cost expenditure for the current study. Future researchers might consider longitudinal data for comparing results to understand the causal effects better. Lastly, conducting an experiment is an avenue for future research because this study is limited to field studies.

## Data Availability Statement

The original contributions presented in the study are included in the article/supplementary material, further inquiries can be directed to the corresponding author.

## Ethics Statement

Ethical review and approval was not required for the study on human participants in accordance with the local legislation and institutional requirements. The patients/participants provided their written informed consent to participate in this study.

## Author Contributions

Conceptualization: HA, AM, and AA. Methodology: AM, AA, and AI. Software: HA and AM. Formal analysis: AM and AI. Writing—original draft preparation: HA, AM, AA, and AI. Writing—review and editing: AM, AA, and AI.

## Conflict of Interest

The authors declare that the research was conducted in the absence of any commercial or financial relationships that could be construed as a potential conflict of interest.

## Publisher's Note

All claims expressed in this article are solely those of the authors and do not necessarily represent those of their affiliated organizations, or those of the publisher, the editors and the reviewers. Any product that may be evaluated in this article, or claim that may be made by its manufacturer, is not guaranteed or endorsed by the publisher.
